# Supplementary material for: Hebbian priming of human motor learning
Source: Nat Commun. 2024 Jun 15;15:5126. doi: 10.1038/s41467-024-49478-5 (PMC11180091; doi:10.1038/s41467-024-49478-5)
Supplement: Supplementary file 1 — Supplementary Information [file 41467_2024_49478_MOESM1_ESM.pdf]

Supplementary information

Hebbian priming of human motor learning

Jonas Rud Bjørndal[1*], Mikkel Malling Beck[1,2], Lasse Jespersen[1], Lasse Christiansen[2,3], Jesper Lundbye-Jensen[1*]

[1] *Movement & Neuroscience, Department of Nutrition, Exercise and Sports (NEXS), University of Copenhagen, Nørre Allé 51, 2200 Copenhagen N, Denmark*

[2] *Danish Research Centre for Magnetic Resonance, Centre for Functional and Diagnostic Imaging and Research, Copenhagen University Hospital Amager and Hvidovre, Kettegård Allé 30, 2650 Hvidovre, Denmark*

[3] *Department of Neuroscience, Faculty of Health and Medical Sciences, University of Copenhagen, Blegdamsvej 3B, 2200 Copenhagen N, Denmark*

Supplementary Methods

The experimental setup for the applied paired corticospinal-motoneuronal stimulation (PCMS) protocols are illustrated in supplementary Fig. 1. Transcranial magnetic stimulation (TMS) and electrical peripheral nerve stimulation (PNS) was delivered while participants were at rest. During the PCMS protocol, TMS and PNS stimulations were temporally paired. Measures of corticospinal excitability were obtained through application of single-pulse TMS while measures of M- and F-wave characteristics were obtained through application of single-pulse PNS. Electromyography (EMG) was recorded from m. FDI of the participants' right hand through surface electrodes (Ag-AgCl, 1 cm diameter, AMBU). In Experiment III, the stimulation protocols targeted the corticospinal-motoneuronal synapses with different inter-arrival intervals between the descending TMS volley and the antidromic volleys in the α-motoneurons evoked by PNS. The interstimulus intervals were based on calculations of individual peripheral and central conduction times calculated from latencies of evoked responses (MEP, M_{max} and F-wave) on each test day for each participant (Supplementary Fig.1).

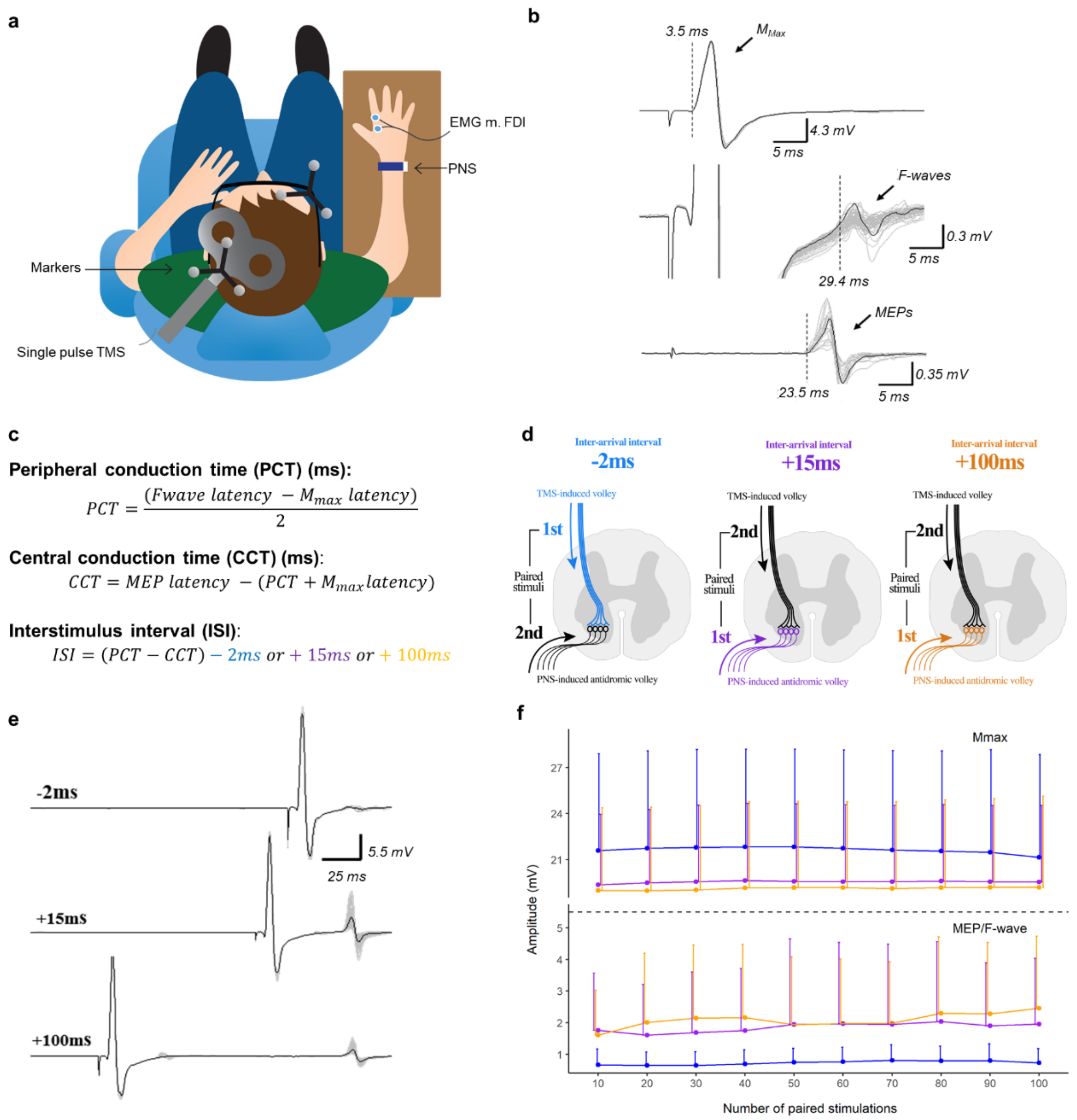

Supplementary Figure 1. Plasticity-inducing protocols. **a)** Experimental setup **b)** Individual latencies from one participant: raw traces of M_{max} (n=60 frames), F-waves (n=60 frames) and MEP during active contraction 10%MVC (n=10 frames) were obtained on each test day for each participant. **c)** Interstimulus intervals were based on calculations of individual peripheral and central conduction times. **d)** The stimulation protocols targeted the corticospinal-motoneuronal synapses with different inter-arrival intervals between the descending TMS volley and the antidromic volleys in the α-motoneurons evoked by PNS stimulation. **e)** Visualizing the different intervals with overdraw traces (n=100 paired stimulations) from each PCMS protocol (-2ms, +15ms and +100ms) from a single participant. **f)** Graph shows the pooled data of M_{max} and MEPs during the PCMS protocols; -2ms (blue), +15ms (purple), and +100ms (orange). Each data point represents the average of 10 traces (M_{max} and MEP's). Note the separation of the y-axis. Error bars indicate SD.

Supplementary Notes

Changes in muscle activity during ballistic motor practice

During the ballistic index finger flexions, EMG was recorded from m. FDI. Raw traces of acceleration and rectified EMG are shown in Supplementary Figure 2a. Since acceleration data showed similar trends in Experiment I and II, we chose to pool data for EMG analyses from the two experiments post hoc (N=46 participants), allowing us to investigate effects of motor practice on muscle activity and to compare PCMS against Control (Rest & Sham). In line with previous studies using ballistic motor learning tasks, we chose to analyze EMG root-mean square amplitude [1,2], as well as the rate of EMG rise (an indirect measure of efferent neural drive)[3] for ballistic trials during baseline measurements and during block 1-3 of motor practice. For EMG_{RMS} amplitude (Supplementary Fig. 2b), the linear mixed model showed a significant main effect of TIME ($F_{(3,7178.9)}=12.16$, $p<0.001$), and a GROUP x TIME interaction ($F_{(3,7178.9)}=6.09$, $p<0.001$). Within TIME, comparisons showed a significant increase from practice Block 1 to practice Block 2 (2.7%±0.9, p=0.03) and from practice Block 1 to practice Block 3 (4.1%±0.9, p<0.01). Pairwise comparisons showed that PCMS led to a significantly larger increase in EMG_{RMS} amplitude from practice Block 1 to practice Block 2 (PCMS: 4.2%±1.3 vs. Control: 1.1%±1.4, p=0.048). A second linear mixed model, only included data from experiment II, was set up to account for unequal amount of data points between day 1 and day 7. At day 7 both groups (PCMS vs. Sham) had maintained their EMG relative to baseline with no significant between-group difference (PCMS: 9.8%±3.7 vs. SHAM: 10.6%±4.1, p=0.92).

For Rate of EMG rise (Supplementary Fig. 3d), the linear mixed model also showed a significant main effect of TIME ($F_{(3,7178.9)}=2.54$, $p=0.050$) and a significant GROUP x TIME interaction ($F_{(3,7178.9)}=12.0$, $p<0.001$). Within TIME, comparisons showed an increase from practice Block 1 to practice Block 2, that did not reach level of significance after correcting for multiple comparisons (4.16%±1.66, p=0.059). Pairwise comparisons showed that PCMS led to a significantly larger increase in rate of EMG rise from practice Block 1 to Block 2 (PCMS: 10.5%±2.2 vs. Control: -2.2%±2.4, p<0.01). At Day 7 we found no significant between-group difference relative to baseline (PCMS: 12.2%±6.9 vs. SHAM: 15%±6.3, p=0.41). Note that a separate model was set up for day 7, since these results only include data from Experiment II. Finally, we investigated whether practice-induced changes in EMG measures correlated with

practice-induced changes in peak acceleration. Pearson correlation tests showed a non-significant correlation between peak acceleration (%baseline) and EMG_{RMS} amplitude (%baseline) (r=0.21, p=0.16) and a significant positive correlation between the rate of EMG rise (%baseline) (r=0.42, p<0.01) and acceleration (%baseline) (Supplementary Fig. 2e). Collectively, these analyses demonstrate a positive effect of ballistic motor practice on FDI muscle activity and larger increases in EMG for PCMS compared to controls. Finally, the results demonstrate that the change in rate of EMG rise for FDI was related to behavioral changes in ballistic motor performance.

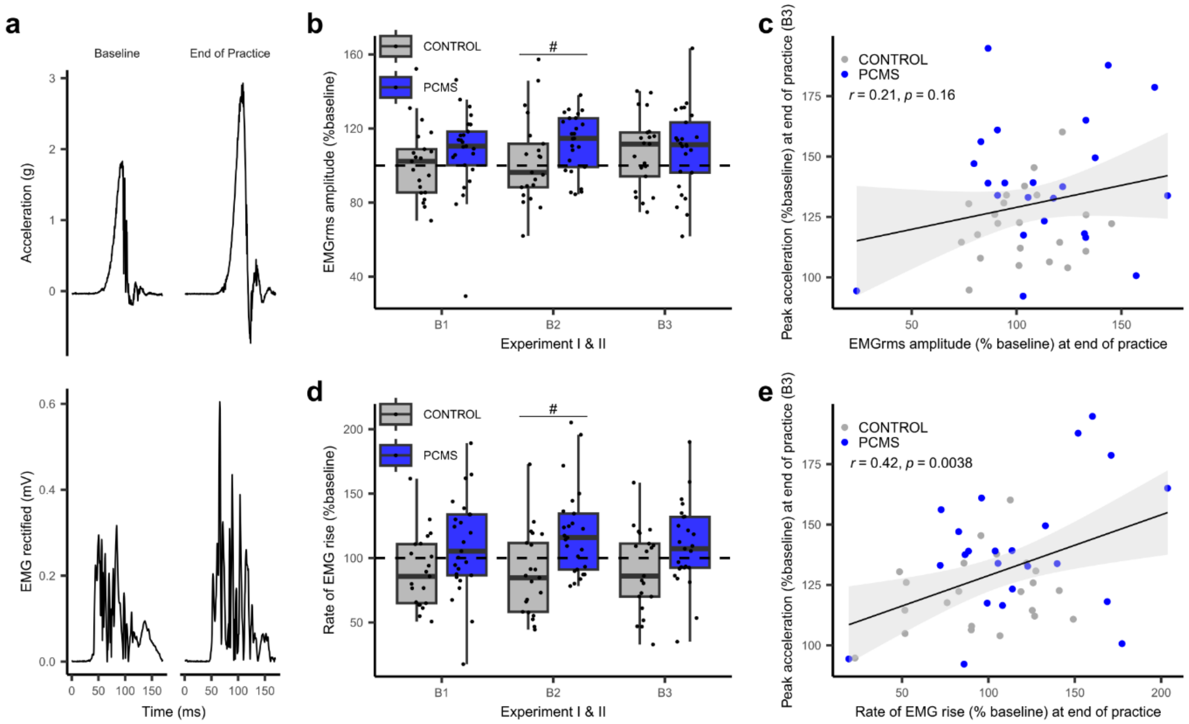

Supplementary Figure 2. Change in EMG during rapid index finger flexions. **a)** Exemplary single subject data, single raw traces of acceleration and EMG (rectified) during the rapid finger flexion. **b)** EMG_{RMS} amplitude analysis from Experiment I-II (n=46 participants). Mean EMG_{RMS} amplitude (% baseline), for both condition PCMS (blue) and Control (grey). The boxplot show the median as the midline, the box bounds the 25th and 75th quartiles and the whiskers bound the minimum and maximum values excluding outliers (defined as values >1.5xinterquartile range), (same boxplot definition are used in (**d**)), single data points represent individual means. The linear mixed model showed a significant GROUP x TIME interaction ($F_{(3,7178.9)}$=6.09, p<0.001). Pairwise comparisons showed that PCMS led to a significantly larger increase in EMG_{RMS} amplitude from practice Block 1 to practice Block 2 (#, p=0.048). **c)** Pearson correlation between EMG_{RMS} amplitude (%baseline) and peak acceleration from the last 10 trials of practice relative to baseline (r=0.21, p=0.16). **d)** Rate of EMG rise analysis from Experiment I-II (n=46 participants). Mean rate of EMG rise (% baseline), for both condition PCMS (blue) and Control (grey). The linear mixed model also showed a significant GROUP x TIME interaction ($F_{(3,7178.9)}$=12.0, p<0.001). Pairwise comparisons showed that PCMS led to a significantly larger increase in rate of EMG rise from practice Block 1 to Block 2 (#, p<0.01). **e)** Pearson correlation between rate of EMG rise (%baseline) and peak acceleration from the last 10 trials of practice relative to baseline (r=0.42, p=0.0038). Source data are provided as a SourceData file.

M_{max} amplitudes and F-wave characteristics

Supplementary Figure 3 illustrates amplitudes of evoked M_{max} responses, amplitudes of evoked F-waves and F-wave persistence during Experiment I, II and III. Statistical analyses of M_{max} and F-wave amplitudes demonstrated no significant main effects in either of the three experiments. For F-wave persistence, we found a main effect of TIME in all three experiments (Experiment I, $F_{(2,28)}=3.83$, $p=0.03$; Experiment II, $F_{(2,39)}=23.9$, $p<0.001$; Experiment III, $F_{(2,144)}=19.9$, $p<0.001$). In Experiment I, we found a significant drop from baseline to post practice (9.5%±3.8, p=0.04). In Experiment II we found a significant drop from baseline to post stimulation (-11.7%+-1.8, p<0.001) and post practice (-8.7%+-1.89, p<0.001). In Experiment III, we found a significant drop from baseline to post stimulation (-12.9%+-2.11, p<0.001) and post practice (-7.0%+-2.11, p<0.01).

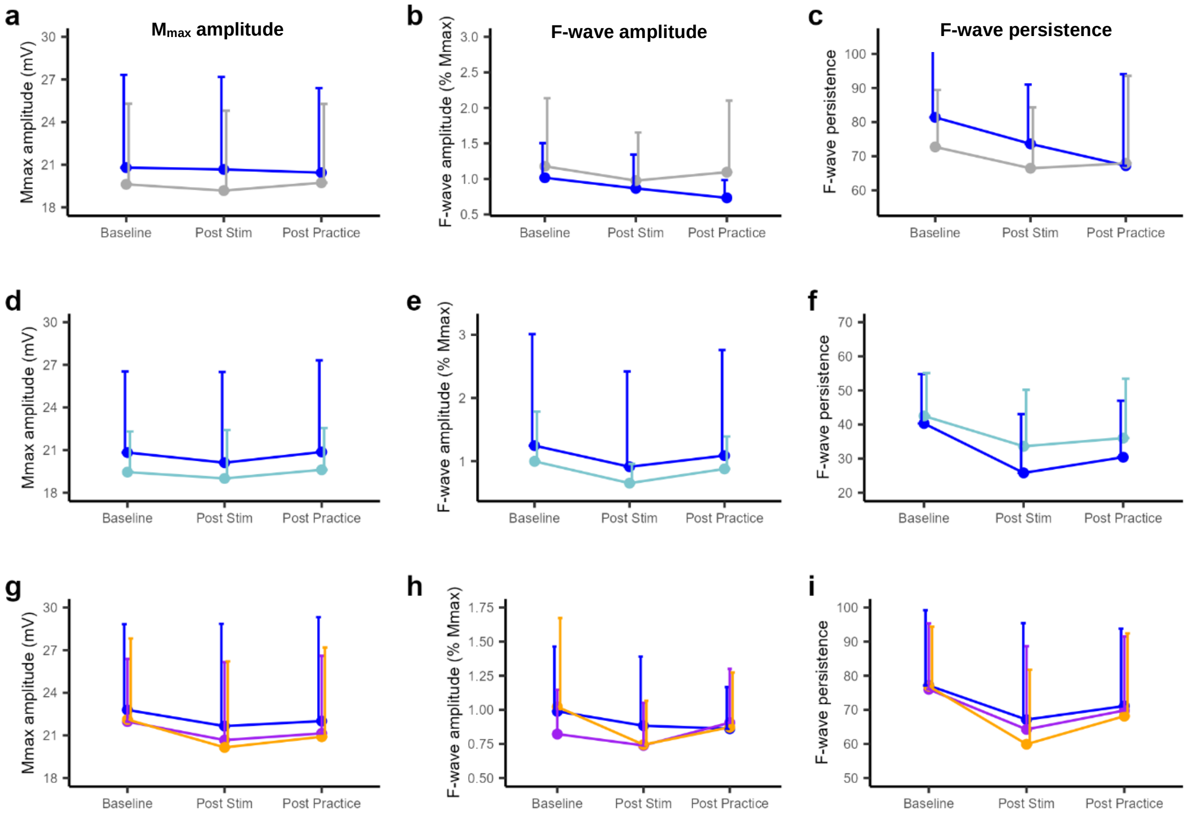

Supplementary Figure 3. Effects of PCMS and motor practice on M_{max} amplitude and F-wave characteristics. Data from **Experiment 1 (a-c**) (n=13 participants in each group) with PCMS (blue) and Rest (grey). **Experiment II (d-f)** (n=10 participants in each group) with PCMS (blue) and Sham (lightblue). **Experiment III (g-i)** (n=18 participants in each group) with PCMS+ (blue), PCMS- (purple) and $PCMS_{coupled\text{-}control}$ (orange). M_{max} amplitude (mV) are shown for all three experiments in a, d, g. F-wave amplitude (% Mmax) is shown in b, e, h. F-wave persistence is shown in c, f, i. Each data point is the group mean with error bars indicating standard deviation. Linear mixed models were used to assess effects of Protocol and Time, For F-wave persistence, we found a main effect of TIME in all three experiments (Experiment I, $F_{(2,28)}=3.83$, p=0.03; Experiment II, $F_{(2,39)}=23.9$, p<0.001; Experiment III, $F_{(2,144)}=19.9$, p<0.001).

Supplementary Discussion

Responders/Non-Responders in PCMS-

MEP amplitudes during PCMS- have previously been proposed as a marker for electrophysiological responders and non-responders to inhibitory PCMS-. A study showed that only individuals in whom, the afferent input reduced MEP size during inhibitory PCMS also displayed suppressed corticospinal excitability after inhibitory PCMS[4]. In the present study, only 6 out of 18 participants displayed decreased MEP amplitudes after the PCMS- protocol. Participants were binary categorized as responders to PCMS- if MEP amplitudes were decreased post PCMS- (Supplementary Fig. 4a). Visually, data shows that participants with decreased MEPs after PCMS- (responders) displayed smaller MEP amplitudes during the PCMS- protocol (even relatively to baseline that was acquired at a lower stimulation intensity than used during PCMS: 120% RMT vs. 150% RMT, respectively) (Supplementary Fig. 4a-c). It could further be speculated that MEP amplitudes during PCMS- in those responding with increases in MEP amplitudes following PCMS- (non-responders) were close to MEP_{max} (this is likely, based on the TMS intensity during PCMS, 150% rMT)[5]. These findings support that MEP amplitudes during PCMS- could be seen as a marker for responders and non-responders, corroborating previous observations[4]. Experiment III showed that PCMS- suppressed early learning, assessed as performance during practice block 1, compared to PCMS+ and $PCMS_{coupled\text{-}control}$. We speculated whether the electrophysiological marker of responsiveness to PCMS- could also explain differences in changes in ballistic motor performance during the early learning phase (practice block 1). However, this did not seem be the case (Supplementary Fig. 4d). This suggests that other mechanisms in addition to a ‘pure’ corticomotoneuronal STDP rule influence the electrophysiological after-effects of PCMS.

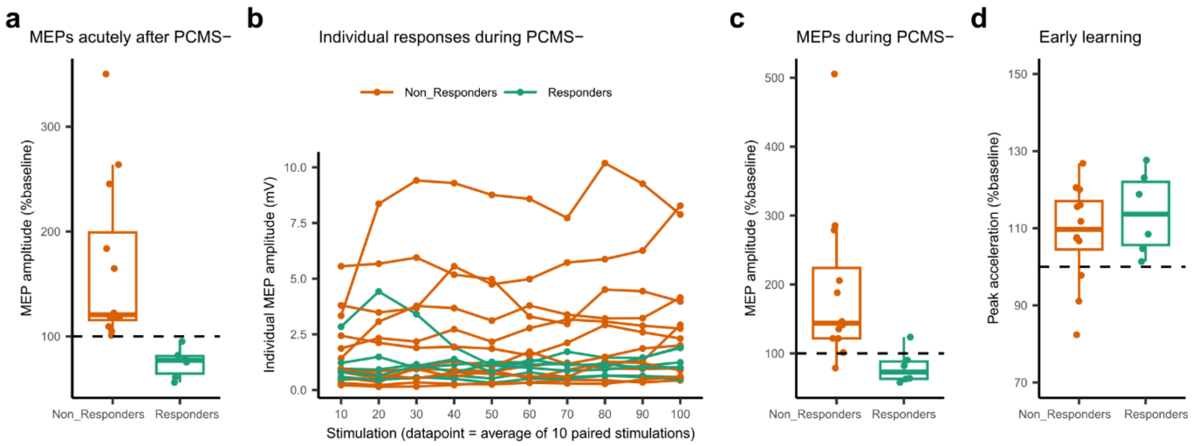

Supplementary Figure 4. Responders vs Non-responders to PCMS-. a) MEP amplitudes post PCMS- (%baseline). 12 participants displayed increased MEP after PCMS- (here categorized as non-responders), and only 6 participants with decreased MEP (responders). The boxplot show the median as the midline, the box bounds the 25th and 75th quartiles and the whiskers bound the minimum and maximum values excluding outliers (defined as values >1.5xinterquartile range), (same boxplot definition are used in (**c-d**)), single data points represent individual MEP means (n=20 MEPs). **b)** MEP amplitudes during PCMS-, divided in whether participants responded as intended to the PCMS- (decreased MEP amplitude post PCMS-). Each datapoint is the individual mean of 10 stimulations. **c)** Group and individual means of MEP amplitudes during all stimulations delivered during PCMS- presented as % of MEP amplitudes at baseline. Please note that for the majority of individuals responding as intended to the PCMS-, the MEP amplitudes delivered during PCMS- (150% RMT) were smaller than MEP amplitudes delivered at baseline (120% RMT) showing an effective reduction of MEP amplitudes. **d)** Responders and non-responders showed similar motor performance, peak acceleration (% of baseline), during the first practice block, each datapoint is the individual mean of the 50 trials in block 1. Source data are provided as a SourceData file.

An alternative mechanism for the contrasting finding that PCMS- impair early learning and increase corticospinal excitability could be the result of single-pulse TMS and PCMS not targeting parts of the same motoneuronal pool. A single TMS pulse with intensity of 120% rMT was used to record MEP's before and after PCMS. It can be assumed that an MEP evoked with this intensity involves activation of approximately half of the motoneuronal pool that can be excited by TMS during rest [5]. In PCMS-, the high-intensity TMS pulse (150% rMT) is closer to the maximum level and can therefore be expected to reflect a substantially larger activation of the motoneuronal pool. The timely paired PNS is supramaximal and antidromic volleys can be expected in all fibers of the stimulated nerve. However, the antidromic activation of the motoneurons favor the larger motoneurons due to collision between H-waves and antidromic activity in the smaller, which further decouples the affected motoneuronal pool from the pool excited by single pulse TMS at 120%. In contrast, movement at maximal speed or with maximal rate of force development depend on both high rate of motor unit recruitment and motoneuronal firing rates[6]. In this light, it is possible that the ability to increase recruitment rates with ballistic motor practice was hampered due to the inhibitory priming that affected corticomotoneuronal synapses on motoneurons that are recruited during maximal ballistic contractions but less so from descending activity evoked when stimulating M1 120 % of rMT.

Lastly, it is relevant to address that the inhibitory effects of the PCMS- (15ms inter-arrival interval at CM-synapse) on corticospinal transmission have been demonstrated in fairly few individuals across studies[4,7,8], probably due to the limited clinical potentials compared to the PCMS+ protocol. We observed that PCMS protocols can affect motor learning. Specifically, we observed that PCMS+ had a positive effect on motor learning, and that PCMS- suppressed early learning. We therefore argue that PCMS+ combined with motor practice are more beneficial and has the largest translational potential in rehabilitation settings.

Supplementary References

1. Rogasch, N. C., Dartnall, T. J., Cirillo, J., Nordstrom, M. A. & Semmler, J. G. Corticomotor plasticity and learning of a ballistic thumb training task are diminished in older adults. *J. Appl. Physiol.* **107**, 1874–1883 (2009).
2. Giesebrecht, S., Duinen, H. Van, Todd, G., Gandevia, S. C. & Taylor, J. L. Training in a ballistic task but not a visuomotor task increases responses to stimulation of human corticospinal axons. *J. Neurophysiol.* **107**, 2485–2492 (2012).
3. Aagaard, P., Simonsen, E. B., Andersen, J. L., Magnusson, P. & Dyhre-Poulsen, P. Increased rate of force development and neural drive of human skeletal muscle following resistance training. *J. Appl. Physiol.* **93**, 1318–1326 (2002).
4. Urbin, M. A., Ozdemir, R. A., Tazoe, T. & Perez, M. A. Spike-timing-dependent plasticity in lower-limb motoneurons after human spinal cord injury. *J. Neurophysiol.* **118**, 2171–2180 (2017).
5. Devanne, H., Lavoie, B. A. & Capaday, C. Input-output properties and gain changes in the human corticospinal pathway. *Exp. Brain Res.* **114**, 329–338 (1997).
6. Del Vecchio, A. *et al.* You are as fast as your motor neurons: speed of recruitment and maximal discharge of motor neurons determine the maximal rate of force development in humans. *J. Physiol.* **597**, 2445–2456 (2019).
7. Taylor, J. L. & Martin, P. G. Voluntary motor output is altered by spike-timing-dependent changes in the human corticospinal pathway. *J. Neurosci.* **29**, 11708–11716 (2009).
8. Bunday, K. L. & Perez, M. A. Motor recovery after spinal cord injury enhanced by strengthening corticospinal synaptic transmission. *Curr. Biol.* **22**, 2355–2361 (2012).
