# Supplementary material for: Hebbian priming of human motor learning
Source: Nat Commun. 2024 Jun 15;15:5126. doi: 10.1038/s41467-024-49478-5 (PMC11180091; doi:10.1038/s41467-024-49478-5)
Supplement: Supplementary file 3 — Reporting summary [file 41467_2024_49478_MOESM3_ESM.pdf]

Corresponding author(s): Jonas Rud Bjørndal
Jesper Lundbye-Jensen

Last updated by author(s): May 15, 2024

# Reporting Summary

Nature Portfolio wishes to improve the reproducibility of the work that we publish. This form provides structure for consistency and transparency in reporting. For further information on Nature Portfolio policies, see our Editorial Policies and the Editorial Policy Checklist.

## Statistics

For all statistical analyses, confirm that the following items are present in the figure legend, table legend, main text, or Methods section.

| n/a | Confirmed | |
|---|---|---|
| ☐ | ☒ | The exact sample size (*n*) for each experimental group/condition, given as a discrete number and unit of measurement |
| ☐ | ☒ | A statement on whether measurements were taken from distinct samples or whether the same sample was measured repeatedly |
| ☐ | ☒ | The statistical test(s) used AND whether they are one- or two-sided<br>*Only common tests should be described solely by name; describe more complex techniques in the Methods section.* |
| ☐ | ☒ | A description of all covariates tested |
| ☐ | ☒ | A description of any assumptions or corrections, such as tests of normality and adjustment for multiple comparisons |
| ☐ | ☒ | A full description of the statistical parameters including central tendency (e.g. means) or other basic estimates (e.g. regression coefficient) AND variation (e.g. standard deviation) or associated estimates of uncertainty (e.g. confidence intervals) |
| ☐ | ☒ | For null hypothesis testing, the test statistic (e.g. *F*, *t*, *r*) with confidence intervals, effect sizes, degrees of freedom and *P* value noted<br>*Give P values as exact values whenever suitable.* |
| ☒ | ☐ | For Bayesian analysis, information on the choice of priors and Markov chain Monte Carlo settings |
| ☒ | ☐ | For hierarchical and complex designs, identification of the appropriate level for tests and full reporting of outcomes |
| ☐ | ☒ | Estimates of effect sizes (e.g. Cohen's *d*, Pearson's *r*), indicating how they were calculated |

*Our web collection on statistics for biologists contains articles on many of the points above.*

## Software and code

Policy information about availability of computer code

| | |
|---|---|
| Data collection | The behavioral task was presented by MATLAB (v. R2021b).<br>Transcranial magnetic stimulation (TMS) were elicited thorugh monophasic single-pulse TMS applied to the contralateral primary motor cortex of the dominant hand via a figure-of-eight TMS coil (Magstim(R)D70^2 connected to a Magstim200). Electrical stimulation with high voltage electrical current (200 µs pulse duration, DS7A; Digitimer) was delivered to the ulnar nerve at the wrist (Bar Stimulating Electrode, Digitimer) to measure the maximal compound muscle action potential (Mmax) and F-waves. Signal software (Cambridge Electronic Design: v6.05) used to record evoked potentials elicited by Transcranial magnetic stimulation (TMS) and peripheral nerve stimulation. Spike2 software (Cambride Electronic Design: v7.10) was used to record electromyography (EMG), position and acceleration during the motor task. A neuro-navigation system (Brainsight 2, Rogue Research, Montreal, Canada) was used to ensure stable positioning of the coil throughout the experiments. |
| Data analysis | 1) R software was used for statistical analysis (v4.1.3)<br>2) R-package ggplot2 (v3.3.5) used for data visualization<br>3) R-package lme4 (v1.1-28)) used to fit linear mixed effect models<br>4) R-package lmerTest(v3.1-3) was used to evaluate main and interaction effects<br>5) R-package multcomp(v1.4-18) was used for pairwise comparisons |

For manuscripts utilizing custom algorithms or software that are central to the research but not yet described in published literature, software must be made available to editors and reviewers. We strongly encourage code deposition in a community repository (e.g. GitHub). See the Nature Portfolio guidelines for submitting code & software for further information.

## Data

Policy information about availability of data

All manuscripts must include a data availability statement. This statement should provide the following information, where applicable:

- Accession codes, unique identifiers, or web links for publicly available datasets
- A description of any restrictions on data availability
- For clinical datasets or third party data, please ensure that the statement adheres to our policy

Data availability statement:
The raw data are protected and are not available due to data privacy laws. The processed source data generated in this study have been deposited in the Figshare database with the following DOI: https://doi.org/10.6084/m9.figshare.23689119. The complete dataset used in the study is available upon request to J.R.B. or J.L.J.

Code availability statement:
Matlab scripts for the custom-made non-commercialized behavioral task are uploaded to Github, see link https://doi.org/10.5281/zenodo.11203521. R-scripts to reproduce figures are uploaded to Github: https://github.com/JONAS-RUD-BJORNDAL/HebbianPrimingMotorLearning.

## Research involving human participants, their data, or biological material

Policy information about studies with human participants or human data. See also policy information about sex, gender (identity/presentation), and sexual orientation and race, ethnicity and racism.

| | |
|---|---|
| Reporting on sex and gender | Both males and females volunteered and provided consent after thorough information of the study procedures. The findings from all four experiments apply to both sex. Sex of participants was determined based on self-report.<br>We did not perform sex specific analyses since all analyses were performed on experimental group levels. All groups included both males and females.<br>Experiment 1: n=26 (14male/12female, mean age 24,9 standard deviation 2.2)<br>Experiment 2: n=20 (10male/10female, mean age 25.5 standard deviation 2.3)<br>Experiment 3: n=18 (9male/9female, mean age 25.3 standard deviation 2.3)<br>Experiment 4a: n=10 (4male/6female, mean age 25.5, standard deviation 2.1)<br>Experiment 4b-c: n=8 (5male/3female, mean age 25.7, standard deviation 2.1) |
| Reporting on race, ethnicity, or other socially relevant groupings | Race, ethnicity, and other socially relevant groupings were not considered and are not reported on in this study design. |
| Population characteristics | Population characteristics: Young adults (aged 20-30 years: mean 25 years (standard deviation 2). Participants were defined as able-bodied based on a standardized general eligibility questionnaire, with no history of neurological, psychiatric or medical diseases and no intake of medication. All participants were right-handed (except one, who had no preference), according to the Edinburgh Handedness Inventory. |
| Recruitment | Participant were recruited from the greater Copenhagen area through physical billboards and Online through social media (Facebook).<br>Potential self-selection bias:<br>- People with an active interest in research might be more likely to volunteer for a research project at the University<br>- Although experiment were performed across the day, to accommodate the time available for the individual participants, it cannot be ruled out that people with a full-time job may have been less likely to volunteer.<br>- Due to the potential influences of these factors, the findings may not be representative for the whole population. |
| Ethics oversight | All experiment procedures were approved by the local ethics committee for the Greater Copenhagen area (Denmark) (protocol: H-17019671), and the study was performed in accordance with the declaration of Helsinki. |

Note that full information on the approval of the study protocol must also be provided in the manuscript.

# Field-specific reporting

Please select the one below that is the best fit for your research. If you are not sure, read the appropriate sections before making your selection.

☒ Life sciences ☐ Behavioural & social sciences ☐ Ecological, evolutionary & environmental sciences

For a reference copy of the document with all sections, see nature.com/documents/nr-reporting-summary-flat.pdf

# Life sciences study design

All studies must disclose on these points even when the disclosure is negative.

| | |
|---|---|
| Sample size | Sample sizes were based on previous studies within the research field that proved sufficient to reach statistical significance with the used |

| | |
|---|---|
| Sample size | techniques and methods also used in the present study. Furthermore, the sample size was tested a priori in R statistical software, based on a significance level of 0.05 and a statistical power of 80%. . |
| Data exclusions | Recordings of motor evoked potentials (MEP) were measured during rest. We observed resting EMG during experiments, any stimulations performed while the participant had muscle activity (above resting levels) were excluded from analysis. |
| Replication | The findings from Experiment I were replicated in the double-blinded and sham-controlled Experiment II. Findings from Experiment I and II were again replicated in Experiment III. All attempts of replication were succesful regarding the effect of paired corticomotoneuronal stimulations on ballistic motor performance. |
| Randomization | In all three experiments, participants were randomized into experimental groups. |
| Blinding | All participants were blinded to the intervention type. Except the Rest group in Experiment I.<br>Experiment I: Investigators were not blinded to group allocation during data collection or analysis<br>Experiment II: To verify findings from Experiment I, we made the double-blinded study in Experiment II. Here, Investigator A, was aware of allocation and Investigator B was blinded to the allocation. Investigator B performed the data analysis blinded.<br>Experiment III: Investigators were not blinded to group allocation during data collection or analysis.<br>Experiment IIII: Investigators were not blinded to group allocation during data collection or analysis. |

# Reporting for specific materials, systems and methods

We require information from authors about some types of materials, experimental systems and methods used in many studies. Here, indicate whether each material, system or method listed is relevant to your study. If you are not sure if a list item applies to your research, read the appropriate section before selecting a response.

## Materials & experimental systems

| n/a | Involved in the study |
|---|---|
| ☒ | ☐ Antibodies |
| ☒ | ☐ Eukaryotic cell lines |
| ☒ | ☐ Palaeontology and archaeology |
| ☒ | ☐ Animals and other organisms |
| ☒ | ☐ Clinical data |
| ☒ | ☐ Dual use research of concern |
| ☒ | ☐ Plants |

## Methods

| n/a | Involved in the study |
|---|---|
| ☒ | ☐ ChIP-seq |
| ☒ | ☐ Flow cytometry |
| ☒ | ☐ MRI-based neuroimaging |

## Plants

| | |
|---|---|
| Seed stocks | not applicable |
| Novel plant genotypes | not applicable |
| Authentication | not applicable |
